# Engineering Cancer/Testis Antigens With Reversible *S-*Cationization to Evaluate Antigen Spreading

**DOI:** 10.3389/fonc.2022.869393

**Published:** 2022-05-04

**Authors:** Ai Miyamoto, Tomoko Honjo, Mirei Masui, Rie Kinoshita, Hiromi Kumon, Kazuhiro Kakimi, Junichiro Futami

**Affiliations:** ^1^ Department of Interdisciplinary Science and Engineering in Health Systems, Okayama University, Okayama, Japan; ^2^ Department of Cell Biology, Okayama University Graduate School of Medicine, Dentistry and Pharmaceutical Sciences, Okayama, Japan; ^3^ Innovation Center Okayama for Nanobio-targeted Therapy, Okayama University, Okayama, Japan; ^4^ Niimi University, Niimi, Japan; ^5^ Department of Immunotherapeutics, The University of Tokyo Hospital, Tokyo, Japan

**Keywords:** autoantibody, biomarker, protein engineering, cancer-immunity cycle, immune monitoring, cancer/testis antigens

## Abstract

Serum autoantibody to cancer/testis antigens (CTAs) is a critical biomarker that reflects the antitumor immune response. Quantitative and multiplexed anti-CTA detection arrays can assess the immune status in tumors and monitor therapy-induced antitumor immune reactions. Most full-length recombinant CTA proteins tend to aggregate. Cysteine residue-specific *S*-cationization techniques facilitate the preparation of water-soluble and full-length CTAs. Combined with Luminex technology, we designed a multiple *S*-cationized antigen-immobilized bead array (MUSCAT) assay system to evaluate multiple serum antibodies to CTAs. Reducible *S*-alkyl-disulfide-cationized antigens in cytosolic conditions were employed to develop rabbit polyclonal antibodies as positive controls. These control antibodies sensitively detected immobilized antigens on beads and endogenous antigens in human lung cancer-derived cell lines. Rabbit polyclonal antibodies successfully confirmed the dynamic ranges and quantitative MUSCAT assay results. An immune monitoring study was conducted using the serum samples on an adenovirus−mediated REIC/Dkk−3 gene therapy clinical trial that showed a successful clinical response in metastatic castration-resistant prostate cancer. Autoantibody responses were closely related to clinical outcomes. Notably, upregulation of anti-CTA responses was monitored before tumor regression. Thus, quantitative monitoring of anti-CTA antibody biomarkers can be used to evaluate the cancer-immunity cycle. A quality-certified serum autoantibody monitoring system is a powerful tool for developing and evaluating cancer immunotherapy.

## Introduction

Growing evidence shows that many patients with cancer benefit from immunotherapy (1). The immune system can eliminate cancer cells by recognizing cancer antigens expressed in malignant cells. This cancer immunosurveillance concept is now clearly described by a seven-step cancer immunity cycle (2). Most cancers adopt strategies to evade the immune system after a long struggle between malignant cells and the immune system (3–5). Thus, reactivation of the antitumor immune response and upregulation of the cancer-immunity cycle are critical to ensure improved clinical response. Immune checkpoint inhibitors are currently the most promising treatment for upregulating the cancer-immunity cycle; however, their clinical responses vary from patient to patient due to the complexity of tumor-immune interactions (6). Recent analysis of the mechanisms of immune suppression in cancer revealed that different steps in the cancer-immunity cycle by which tumors escape immunosurveillance are likely to differ among patients (7, 8). Therefore, cancer immunotherapy needs to be personalized to identify the rate-limiting steps in individual patients, and a combination of strategies should be used to overcome these hurdles. To realize personalized precision cancer immune therapy, a technique that can monitor the cancer-immunity cycle will be a powerful tool for treatment.

The immunogenicity of cancer cells is determined by antigen peptides present on MHC class I, and CD8^+^ cytotoxic lymphocytes eliminate cells by recognizing this complex (9). Cancer antigens can be classified into two groups: aberrantly expressed tumor-associated antigens (TAAs) (10) and cancer/testis antigens (CTAs) (11, 12), or neoantigens derived from mutated gene products (13, 14). Both TAAs and CTAs are known as shared antigens that are universally detectable in different patients. In contrast, somatic mutation-derived neoantigens show patient-specific individual variations (15).

**Figure 1:**
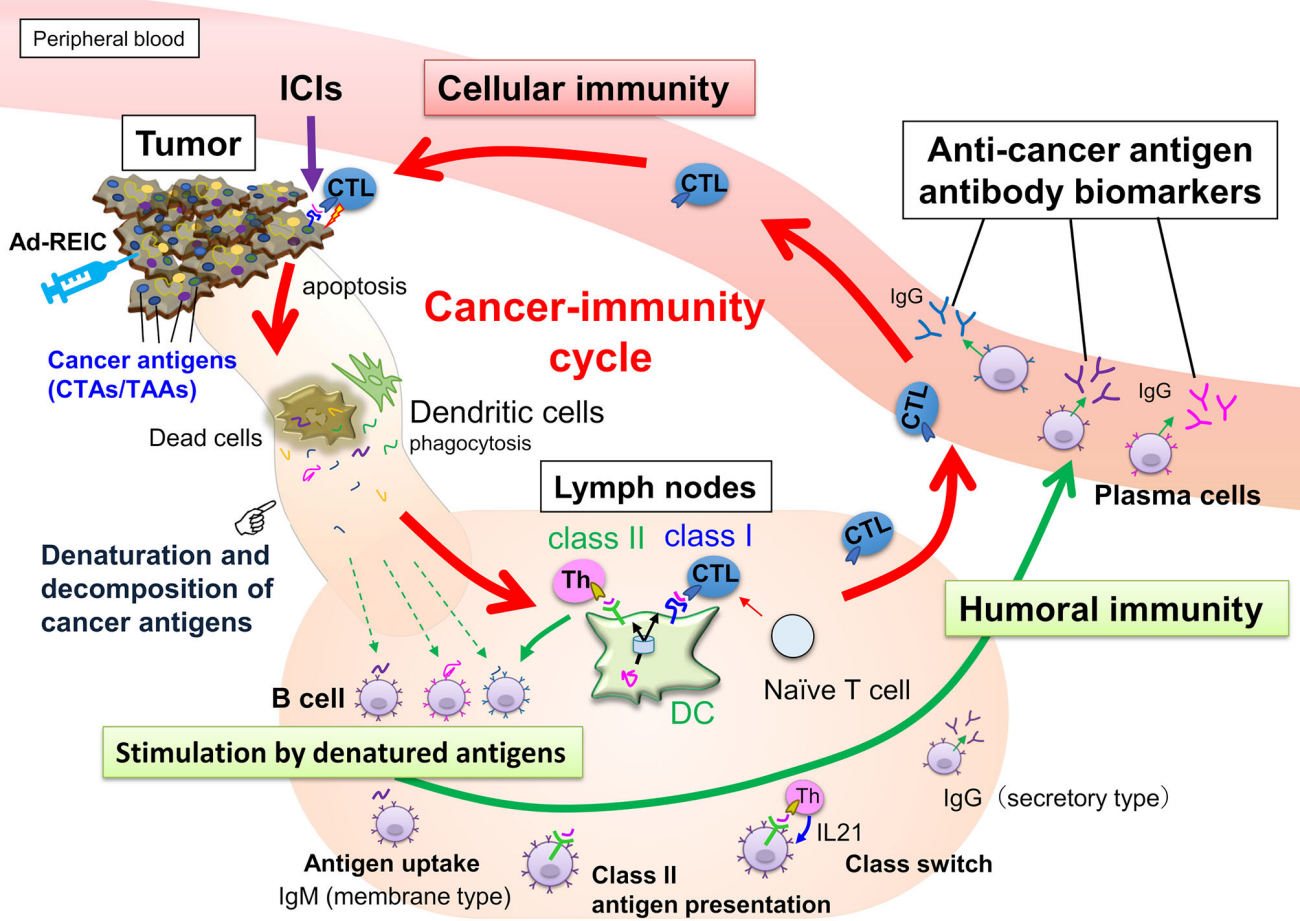
Anticancer immunity is enhanced by the cancer-immunity cycle, and cytotoxic T-lymphocytes (CTLs) eliminate cancer cells. Along with activating the cancer-immunity cycle, antibodies against cancer antigens upregulate by stimulation of released antigens from the cancer cells. Most intracellular cancer antigens suggest easy to denature and decompose after release from cancer cells due to their unstable physical properties. Antibody-producing cells that bind to denatured cancer antigens proliferate, so anti-cancer antigen IgGs recognizing the amino acid sequence of linear epitopes increase preferentially.

During the cancer-immunity cycle activation, antigens released from cancer cells are then captured by dendritic cells, but not all the TAA- or CTA-derived peptides can present on MHC class I. However, these aberrantly expressed antigens could be involved in the humoral immune response. Hence, serum autoantibodies to cancer antigens reflect the current immune response level associated with tumor volume and antigenicity (**Figure 1**). Once cancer cells are destroyed, many cancer antigens are released from them, captured by antigen-presenting cells, and induce T and B cell immune responses. The activation of the antitumor immune response is accompanied by an increase in the number of autoantibodies against various cancer antigens, referred to as antigen spreading (16–22) which could be a critical pharmacodynamic biomarker for the clinical outcome of cancer immunotherapy (18, 22–26). Accumulating evidence indicates that antibody-antigen immune complex uptake through Fcγ receptors on antigen-presenting cells induces cross-presentation, stimulating long-term antitumor cellular immunity (27). Thus, a simple blood test-based evaluation of antigen spreading with cancer-immunity cycle activation could predict systemic cancer immunity.

The diagnosable set of cancer antigens requires a comprehensive array because the expression pattern, antigenicity, and epitopes vary in individual patients (23, 28, 29). Full-length cancer antigen preparation is favored for monitoring antigen spreading based on these requirements. However, many recombinant CTAs appear to show an aggregation-favored unstable property (30). This property is consistent with the bioinformatics prediction of the structure of CTAs, which shows that the majority of CTAs are intrinsically disordered proteins (IDPs) (31). These IDPs, or IDP regions, lack rigid tertiary structures under physiological conditions *in vitro*; however, they can fold after binding to target macromolecules *in vivo (* 32). Thus, recombinant CTA proteins in a disordered conformation frequently form inclusion bodies in host cells (33).

In order to quantitatively evaluate antigen spreading, we designed a multiple *S-*cationized antigen-immobilized bead array (MUSCAT) assay system (33). *S-*cationization techniques are employed as a powerful solubilization tool by conjugation of cationic moieties in sulfhydryl groups in denatured protein (34–36). Full-length and water-soluble *S-*cationized antigens were covalently immobilized onto Luminex magnetic beads *via* the activated carboxylic acid of the COOH radical group (33, 37). Although immobilized *S-*cationized antigen on beads modified all Cys residues and limited amino-groups employed for immobilization, specific antibodies raised in cancer patients were quantitatively detected by the Luminex assay with high sensitivity. This MUSCAT assay system can detect polyclonal antibodies recognizing the linear epitope. Epitope-mapping study of anti-CTA autoantibodies in patient sera revealed that these antibodies are polyclonal and recognize individually different linear epitopes (28). Most CTAs are predicted to have no rigid ordered conformation (31), so antibodies recognizing conformational epitopes are most likely rare. Furthermore, recent knowledge-based and *in silico* analyses of linear epitopes showed that Cys is a minor frequent amino acid residue (38). Chemically modified Cys has minimal effect on the antibody binding efficiency. Thus, the MUSCAT assay system is a powerful strategy for quantifying antigen spreading to diagnose cancer immunotherapy.

Monitoring antigen spreading by the level of autoantibody biomarker requires validated positive control to ensure diagnostic accuracy. This study demonstrated that water-soluble reversibly *S-*cationized CTAs can be employed in both antigens recognized by serum antibodies on the MUSCAT assay and antigens to immunize rabbits to develop antibody-positive controls. Subsequently, this quality-certified serum autoantibody monitoring system also demonstrated successful immune monitoring using clinical samples. In a clinical trial, adenovirus−mediated REIC/Dkk−3 (Ad-REIC) gene therapy has shown a successful clinical response in metastatic castration-resistant prostate cancer (39–41). Ad-REIC is known to induce cancer cell-specific apoptosis (42) and activate the antitumor immune response (43, 44), so this serum sample was used to evaluate the MUSCAT assay system in the current study. It was confirmed that the elevated autoantibody biomarker closely related to the clinical response showed potential to assist clinical decisions by monitoring the level of the cancer-immunity cycle.

## Materials and Methods

### Preparation of Recombinant Antigens

The cDNAs for antigens encoding the full-length and mature form of NY-ESO-1/CT6.1 (Uniprot: P78358), MAGE-A4/CT1.4 (Uniprot: P43358), XAGE-1b/CT12.1 (Uniprot: Q9HD64), MAGE-C2/CT10 (Uniprot: Q9UBF1), DDX53/CT-26 (Uniprot: Q86TM3), WT-1 (Uniprot: J3KNN9), CEP55 (Uniprot: Q53EZ4), LY6K/CT97 (Uniprot: Q17RY6), PSG8 (Uniprot: Q9UQ74), and ZNF165/CT53 (Uniprot: P49910) were cloned into pET28b vectors (Novagen) to express a His-tag (MGSSHHHHHHSSGLVPRGSH) on the N-terminus, and StrepTagII (GPGWSHPQFEK) on the carboxyl terminus. An expression vector for enhanced green fluorescent protein (EGFP) was designed using the same procedure. All recombinant proteins were expressed in *Escherichia coli* BL21(DE3). MAGE-A4, MAGE-C2, and EGFP expressed as soluble fractions were purified by immobilized metal affinity chromatography (IMAC). The other eight recombinant proteins expressed as insoluble inclusion bodies were solubilized by reversible *S-*cationization using [3-(trimethylammonium)propyl]-methanethiosulphonate (TAPS-sulfonate, Katayama Chemical, Osaka, Japan), as described previously (33–35). Antigens, containing degraded impurities, were further purified by a reversed-phase HPLC column (COSMOSIL Protein-R, 4.6 mm I.D. × 150 mm, Nacalai Tesque Inc.) using an acetonitrile linear gradient elution procedure, in the presence of 0.1% HCl (**Supplementary Figure 1**).

### Immunization and Purification of Polyclonal Antibody

Antiserums against ten recombinant antigens were prepared by Cosmo Bio (Tokyo, Japan) by immunizing rabbits with native MAGE-A4 or nine TAPS-antigens. The IgG fraction was precipitated using 40% ammonium sulfate and dialyzed against PBS. The sample was then diluted three times with 60 mM acetate buffer (pH 4.8), and 6.8% caprylic acid was added to precipitate fibrinogen (45). After dialysis against PBS, anti-HisTag and anti-StrepTagII antibodies were captured using HisTag-EGFP-StrepTagII protein-immobilized NHS-sepharose (GE Healthcare). Specific antibodies against each antigen were purified using each antigen-immobilized column from the pass-through fractions described above.

### Cell Culture

Human lung cancer-derived cell lines (NCI-H1299, NCI-H1975, and A549), a cervical cancer-derived cell line (HeLa S3), and an ovarian cancer-derived cell line (SK-OV-3) were obtained from the American Type Culture Collection (Manassas, VA, USA). The cells were maintained at 37°C with 5% CO_2_. All cell lines were cultured in RPMI 1640 medium supplemented with 10% fetal bovine serum and penicillin/streptomycin (Wako, Osaka, Japan). DNA demethylation was demonstrated by the addition of 5 μM 5-aza-2’-deoxycytidine (decitabine, DAC, LC Laboratories, Woburn, MA, USA) to the cell culture (46).

### Western Blot Analysis

Cultured tissue cells were lysed in lysis buffer supplemented with a protease inhibitor cocktail and disrupted on ice using a sonicator. The protein concentration of cell lysates was assessed using the Bradford protein assay (Bio-Rad Laboratories, Hercules, CA, USA) with bovine serum albumin as a standard. Each cell lysate (20 µg) was subjected to SDS-PAGE using a 5-20% gel (Wako, Osaka, Japan) and transferred to a PVDF membrane. After blocking with PVDF-blocking reagent (Toyobo, Osaka, Japan), 1 μg/mL of purified polyclonal antibody for each antigen in Can Get Signal 1 (Toyobo) were incubated with the membrane. Immunoreactive antigens were detected using anti-rabbit IgG HRP-linked antibody (Cell Signaling Technology, Tokyo, Japan) and Western Lightning Plus ECL (PerkinElmer, Waltham, MA, USA). The control monoclonal antibodies to MAGE-A4 (clone: E701U, Cell Signaling Technology, Tokyo, Japan) and XAGE-1b (clone: USO9-13) (47) were employed for the validation of the specificity of polyclonal antibodies. The membrane was reprobed with an anti-β-tubulin antibody (Wako).

### Reverse Transcription (RT)-PCR

Total RNA was isolated from cultured tissue cells using the ISOSPIN Cell & Tissue RNA kit (Nippon Gene, Tokyo, Japan). First-strand cDNA was synthesized from 500 ng of total RNA using PrimeScript™ IV 1st strand cDNA Synthesis Mix (Takara Bio, Shiga, Japan). Gene expression of antigens was evaluated by PCR using primer pairs for NY-ESO-1 (F: 5′- ACATACTGACTATCCGACTGAC-3′; R: 5′- AGGCTGAGCCAAAAACAC-3′), MAGE-A4 (F: 5′-AAACCAGCTATGTGAAAGTCC-3′; R: 5′-ACTCCCTCTTCCTCCTCTAAC-3′), and XAGE-1b (F: 5′-GAGCCCCAAAAAGAAGAACC-3′; R: 5′-GCTCTTGCAGATCACCTTCC-3′). Housekeeping gene expression was confirmed using the PCR primer pair for β-actin (F: 5′-AGAGCTACGAGCTGCCTGAC-3′; R: 5′-AGCACTGTGTTGGCGTACAG-3′).

### Immunostaining of Endogenous CTAs

Sub-confluent cells on a glass-base dish (Iwaki Glass, Shizuoka, Japan) were fixed with 4% paraformaldehyde phosphate buffer solution (Wako) and permeabilized with 0.1% Triton X-100 in PBS for 30 min. Intracellular antigens were reacted with 5 μg/mL of purified polyclonal antibody for each antigen in PBS for 1 h at room temperature. Immunoreacted antigens and nuclei were stained with 2 μg/mL of goat anti-rabbit IgG, Alexa Fluor488 conjugated antibody (Life Technologies), and DAPI (Dojindo Laboratories, Kumamoto, Japan), respectively. Fluorescent images were acquired using a BC43 confocal microscope (Oxford Instruments, Abingdon, UK). Immunostaining of MAGE-C2 in tissue sections was performed using commercially available antibodies (HPA062230, Atlas Antibody, Stockholm, Sweden).

### Validation of Luminex Beads and Beads Assay

Eight TAPS-antigens and two native antigens, certified for their purity, were immobilized to Bio-Plex Pro™ Magnetic COOH Beads (Bio-Rad) designed on a 10-plex assay panel (color-code:#27,35,37,43,45,46,53,55,62,64), according to the manufacturer’s instructions. Beads assay for patient sera and titration assay by affinity-purified polyclonal antibodies designed as a positive control for the 10-plex assay were performed as described previously (33). Briefly, serially diluted antisera in Block Ace (DS Pharma Biomedical, Osaka, Japan) were incubated with 1000 beads for each antigen-immobilized bead in a 96-well microplate (Greiner Bio-One, Tokyo, Japan). After washing with Bio-Plex Pro wash station (Bio-Rad), antibodies on beads were detected by biotin-conjugated, either anti-human IgG or anti-rabbit IgG (Vector Laboratories) and labeled with streptavidin-PE (Vector Laboratories). Analysis was performed with Bio-Plex200 (Bio-Rad), and the mean fluorescence intensity (MFI) was determined from the values for 50 events (beads) per antigen at a minimum.

### Immune Monitoring Study

A 63-year-old man with metastatic castration-resistant prostate cancer, who had shown promising results by Ad-REIC gene therapy, was chosen for the case study (39). Frozen serum samples from a clinical trial on metastatic castration-resistant prostate cancer treated with Ad-REIC (UMIN-CTR ID: UMIN000004929) (39, 40) were used for antibody monitoring. The participants provided written informed consent under institutional review board permission at Okayama University Hospital. The prostate-specific antigen (PSA) level in serum was used from previous data. Control human serum from ten healthy donors (five male and five female, ages 19 to 64), and pooled serum from ten donors (five male and five female, ages 19 to 49) were obtained from Tennessee blood services (TN, USA).

## Results

### Characterization of Rabbit Antibody Immunized by Reversibly *S-*Cationized Antigen

**Figure 2:**
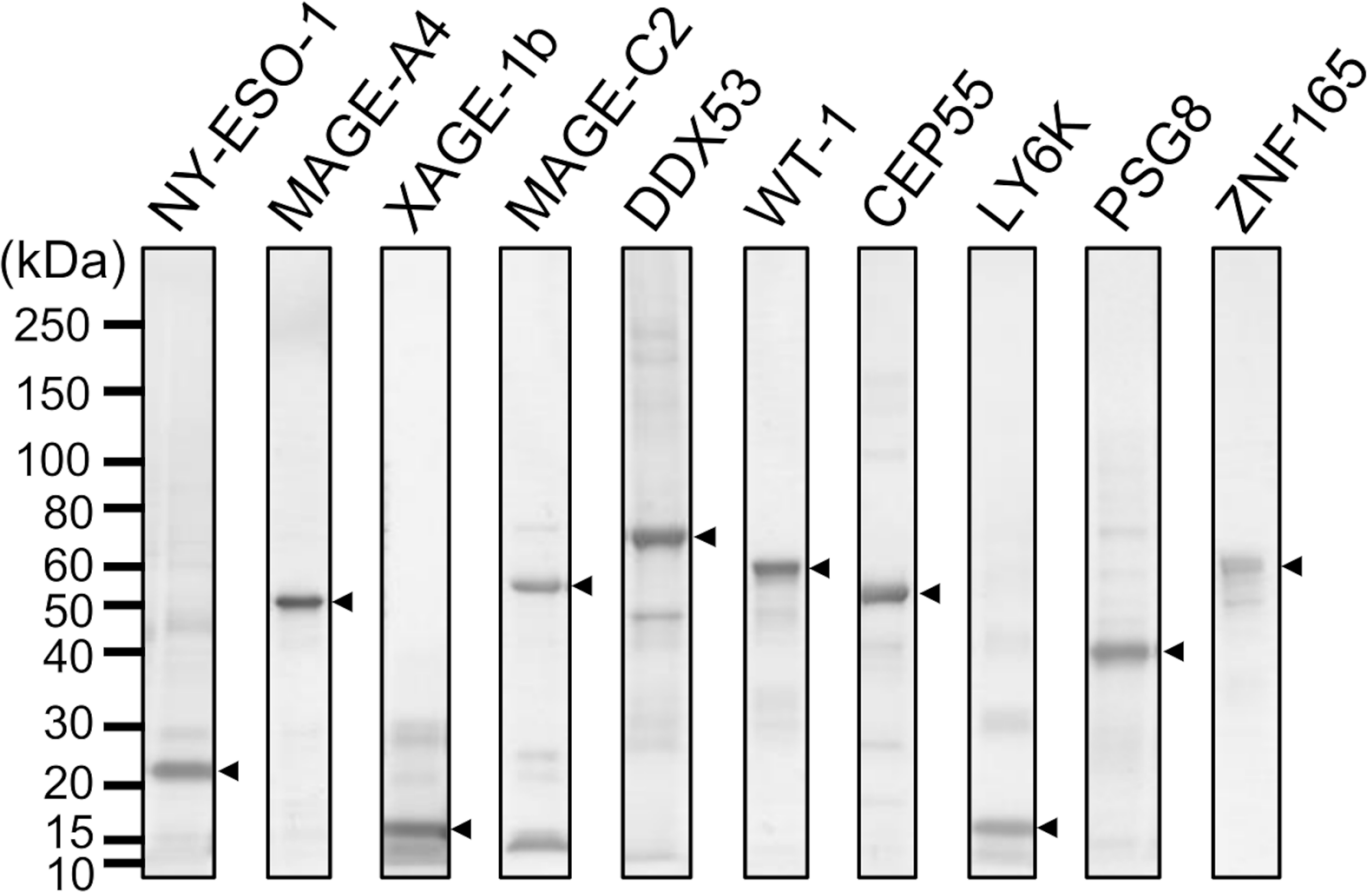
SDS-PAGE analysis of antigens employed for MUSCAT assay.

**Figure 3:**
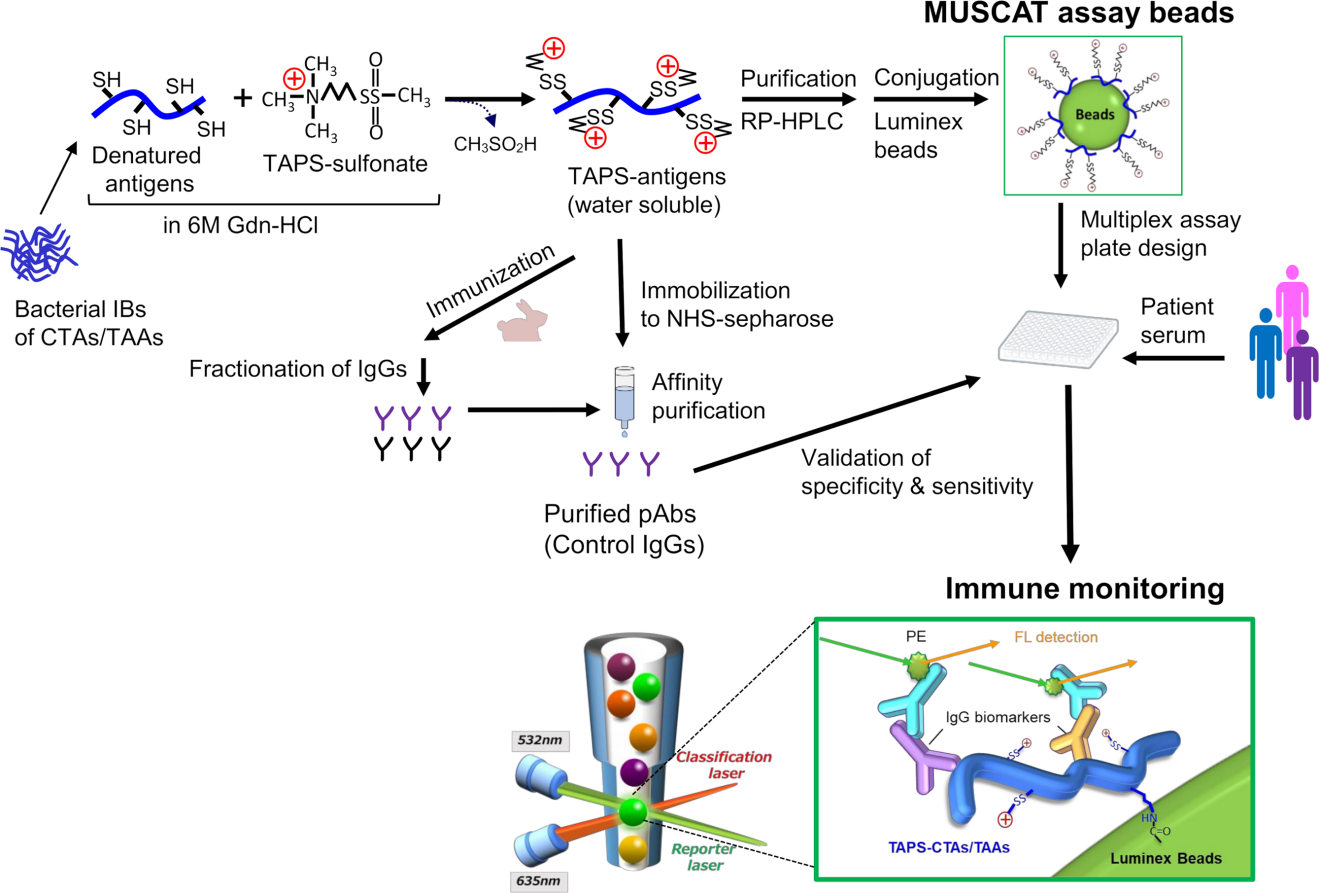
Schematic presentation of antigen engineering of CTAs/TAAs based on solubilization of denatured proteins by *S*-cationization techniques. Water-soluble and full-length TAPS-antigens were employed to capture specific antibodies for immune monitoring and immunization antigens to develop the control IgGs.

**Figure 4:**
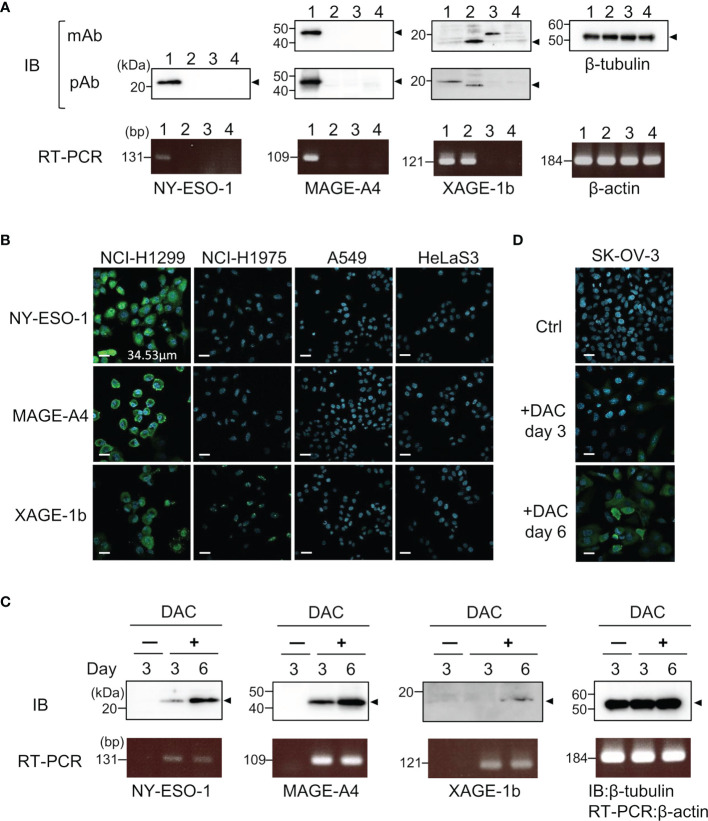
Specific binding of control IgGs developed by TAPS-antigen immunization was confirmed by the binding study of endogenous antigens. **(A)** Detection of intracellular antigens in cell lysates lane 1, NCI-H1299; lane 2, NCI-H1975; lane 3, A549; lane 4, HeLa S3 cells. Cell lysates were analyzed by western blotting with monoclonal antibodies or polyclonal antibodies (upper). mRNA expression levels of each antigen were evaluated by RT-PCR (lower). **(B)** Immunofluorescence staining of NCI-H1299, NCI-H1975, A549, and HeLa S3 cells for nucleus (blue) and intracellular antigens (green). **(C)** Detection of induced CTAs expression protein in SK-OV-3 cells treated with 5 μM DAC. Samples were collected after three or six days of cultivation with DAC. The upper panel is western blotting, the lower panel is RT-PCR. **(D)** Immunofluorescence staining of SK-OV-3 cells treated with 5 μM DAC for nucleus (blue) and intracellular NY-ESO-1 (green).

Specific antibodies recognizing human CTAs or TAAs are useful as positive controls to validate quantitative antibody detection arrays. Most recombinant CTAs/TAAs are expressed as insoluble inclusion bodies in the *E. coli* expression system; *S-*cationization techniques allow for the preparation of water-soluble antigens. Several antigens required purification by reversed-phase HPLC because degraded products were also solubilized during the preparation of *S*-cationized protein (**Supplementary Figure 1**). All recombinant antigens used in this study were verified by SDS-PAGE (**Figure 2**). In this study, the native conformation of MAGE-A4 as well as reversibly *S-*cationized TAPS-NY-ESO-1 and TAPS-XAGE-1b were used to confirm the availability of the antigens for immunization of rabbits (**Figure 3**). These antisera showed high sensitivity to detect the endogenous level of intracellular antigens in western blotting and immunofluorescence staining (**Figures 4A, B**). Although both NY-ESO-1 and XAGE-1b were immunized as alkyldisulfide-modified forms, antibodies raised in rabbits specifically recognized linear epitopes in denatured antigen (**Figure 4A**), as well as epitopes in the native conformation (**Figure 4B**). The antigen specificity between purified polyclonal antibodies and monoclonal antibodies was almost comparable. Antibody response patterns for XAGE-1b are complicated because there are multiple variants (**Supplementary Figure 2**). The nuclear localization of the granular-like pattern of XAGE-1b in NCI-H1975 cells was consistent with previous immunohistochemical results in lung cancer tissues and cells (17, 48). This specific antibody is also detectable in the epigenetically regulated expression of CTAs. SK-OV-3 cells treated with DNA-methylation inhibitor of decitabine (DAC) showed induction of NY-ESO-1, MAGE-A4 and XAGE-1b as determined by measuring mRNA and protein expression levels (**Figures 4C, D**).

### Validation of MUSCAT Assay Panel

**Figure 5:**
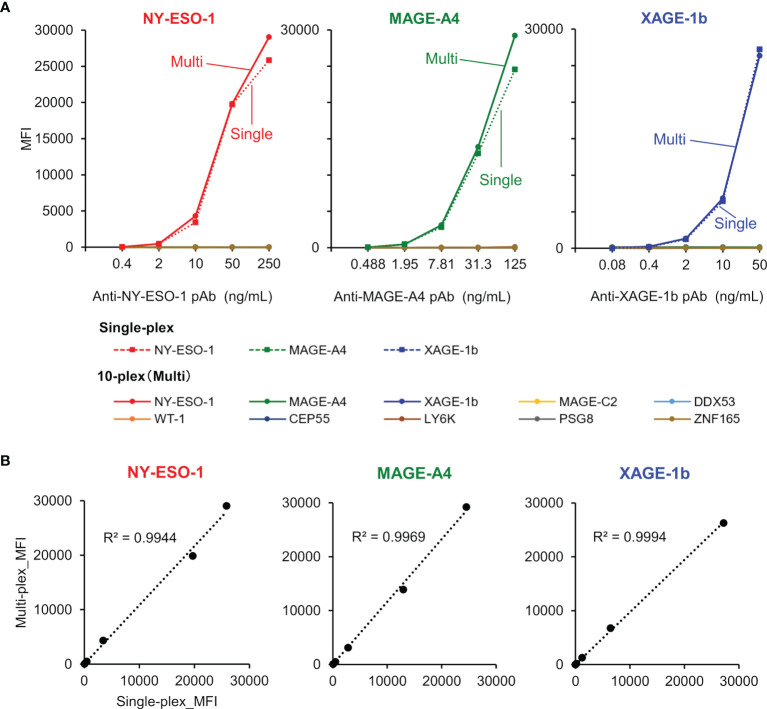
The validation study used purified rabbit polyclonal anti-CTAs antibodies in the single-plex and 10-plex beads assay. **(A)** The solid line represents the 10-plex assay, and the dotted line indicates the single-plex assay. **(B)** Correlation analysis between single-plex and 10-plex assay.

The specific binding of purified rabbit polyclonal anti-CTA antibodies was confirmed using both single-plex and 10-plex bead assays (**Figure 5A**). After purification of each specific antibody using an antigen-immobilized column, linearity and dynamic range of antibody detection in the MUSCAT assay were successfully confirmed in both single and 10-plex assays. The MFI values, calculated from more than 50 beads events in single and 10-plex assays, were highly correlated (**Figure 5B**). The detection ranges for NY-ESO-1(0.32-231 ng/mL), MAGE-A4 (0.12-489 ng/mL), and XAGE-1b (0.08-50 ng/mL) indicated the high sensitivity of these specific antibodies. Although every antigen was designed to possess HisTag and StrepTagII, no cross-reactivity was observed with the purified antibodies. Using this procedure, the preparation of all sets of specific antibodies will be an excellent tool for certifying the specificity and sensitivity of the MUSCAT assay inter-assay or preparation lot.

### Monitoring of Activation of Cancer-Immunity Cycle by Autoantibodies

**Figure 6:**
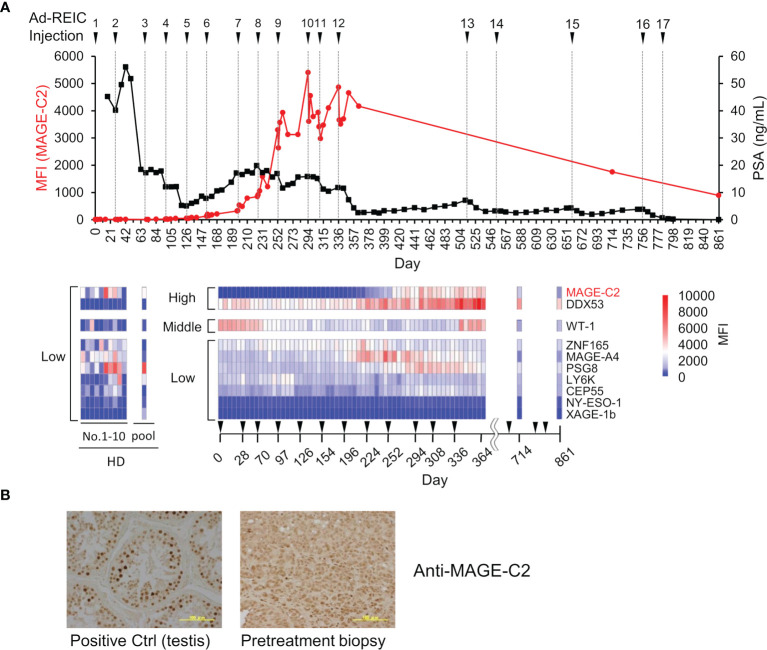
Demonstration of immune monitoring by MUSCAT assay panel on a single, patient-derived clinical sample. **(A)** The line graph represents anti-MAGE-C2 antibodies and PSA changes. The heat map shows changes in the levels of 10 different serum autoantibodies. The dilution ratio of serum autoantibodies was adjusted to 80,000-fold dilution for high titer, 16,000-fold dilution for middle antibody titer, and 1,600-fold for low titer samples. Autoantibody levels in ten healthy donor individuals and a pooled sample. **(B)** Detection of MAGE-C2 protein in the tumor tissues. Scale bar represents 100 µm.

To evaluate the potency of the MUSCAT assay system, changes in serum autoantibody levels were evaluated in one patient successfully treated with Ad-REIC cancer gene therapy (39). Intratumoral injection of Ad-REIC into metastatic lymph nodes induces ER stress-mediated apoptosis by overexpression of REIC, which is then extracellularly secreted where it upregulates immune reactions. Thus, tumor regression is thought to be related to activation of the cancer-immunity cycle. As shown in **Figure 6A**, drastic upregulation of anti-MAGE-C2 and anti-DDX53 was observed during the therapy. Other antibodies for WT-1, ZNF165, MAGE-A4, and PSG8 also increased along with the therapy, typically representing antigen spreading. One of the drastically induced anti-MAGE-C2 antibodies was confirmed to induce immune responses in tumor microenvironments because MAGE-C2 was detected in pretreatment biopsy (**Figure 6B**).

This clinical trial succeeded in complete regression of metastatic castration-resistant prostate cancer by repeating 17 intratumoral injections of Ad-REIC for more than two years (39). Notably, the earlier detection (approximately day 170) of increased autoantibody level before the PSA level decreasing is an important aspect of clinical use of the MUSCAT assay. In addition to the remission of cancer by Ad-REIC, autoantibodies were also reduced. The disappearance of antigen stimulation seems to be closely related to antibody levels. The detection of moderate activation of the cancer-immunity cycle by a small aliquot of a blood sample will be an excellent value for the decision of treatment course.

## Discussion

A recent oncological study revealed that cancer is heterogeneous (49). Heterogeneity in cancer is not only limited to different patients but also occurs within a single patient (50). Certain tumors can be analyzed by molecular profiling yielding clinically relevant diagnostic and prognostic results (49). However, intrapatient or intratumoral heterogeneity remains a significant challenge for deciding the course of clinical cancer treatment. As such, activating the cancer-immunity cycle is a reasonable strategy to overcome this heterogeneity issue (49). The death of the heterogeneous population of cancer cells can be a trigger for priming naïve T cells by newly exposed antigens, resulting in the activation of the cancer-immunity cycle. Antigen exposure from cancer cells also enhances pre-existing memory T and B cells. Upon induction of these immune responses, T cell-mediated cellular immunity targeting various tumor antigens may help regress heterogeneous cancer. Humoral immune responses also increase during the cancer-immunity cycle activation (**Figure 1**); therefore, anti-CTA/TAA IgGs are biomarkers that can reflect this anticancer immunity. The IgG autoantibody biomarker is superior in physicochemical aspects because IgGs in peripheral blood are relatively stable proteins (51). Furthermore, the bias in sampling or storage conditions are nearly negligible.

The preparation of a comprehensive array set of CTAs/TAAs is preferable for reliable evaluation of antigen spreading. Each antigen-specific antibody assay must be validated for its specificity and sensitivity for clinical use. In this study, the water-soluble full-length reversibly *S-*cationized TAPS-antigen was demonstrated to employ both antigens to capture specific antibodies in assay and antigens to develop the antibody in the immunized animals. Purified antibodies reliably validated control in the MUSCAT assay system. Most CTAs have unstable and aggregation-favored properties (30); therefore, *S-*cationization is a powerful technique to prepare highly purified and water-soluble antigens. We are currently preparing comprehensive sets of CTAs/TAAs and each specific antibody for use as a validation tool using these procedures.

The preparation of CTA-specific antibodies also allowed us to gain an understanding of the structural properties of the CTAs (**Figure 4**). TAPS-CTAs are fully denatured, so antibodies immunized by them recognize linear epitopes. However, these antibodies also recognize intracellular endogenous CTAs under non-denaturing conditions. This strongly suggests that the conformation of CTAs has no rigid conformation in cells. CTAs are originally found in testicular cells and are predicted to form a cooperative structure (31). However, aberrantly expressed CTAs in cancer lack this cooperative expression. Therefore, the CTAs released from apoptotic cancer cells are supposed to be easy to denature or decompose. Antibody-producing cells proliferate that bind to cancer antigens released from dead cancer cells; thus, most anti-CTAs bind to the denatured form of protein. A class switch from IgM to IgG also requires antigen binding and cytokine stimulation (52). This mechanism explains why anti-CTAs are recognized preferably in linear epitopes (**Figure 1**).

Many CTAs, including MAGE-A4, NY-ESO-1, and XAGE-1b, are located on the X chromosome and are epigenetically regulated. Lack of X chromosome inactivation in cancer cells induces aberrant epigenetic changes and CTAs expression. Highly immunogenic cancer cells, expressing CTAs, tend to be targeted by the host immune system; thus, suppression of CTAs by DNA methylation could be a mechanism of immune escape in cancer (53, 54). DNA methylation inhibitor treatment, to upregulate the immunogenicity of tumors, would therefore facilitate targeting by the host immune system (55). Ovarian cancer cell line SK-OV-3, has been reported as a model for reactivation of NY-ESO-1 by DAC (56). Increased levels of CTAs after DAC treatment were detected using anti-CTAs antibodies (**Figures 4C, D**). The polyclonal antibodies, immunized full-length CTAs, detected endogenous antigens and their transcript variants. Interestingly, XAGE-1b includes various uncharacterized immune reactive variants (**Supplementary Figure 2**). Determination of these target antigens is crucial for enhancing the accuracy of the immune monitoring system.

As discussed above, both antigen exposure by inducing apoptosis in cancer cells and releasing immune-suppressing machinery is critical to upregulate the cancer-immunity cycle. Intratumoral injections of Ad-REIC therapy are potent in inducing both effects (39). In this therapy, Ad-REIC was injected into the metastatic lymph nodes. This strategy may contribute to the upregulation of the cancer immunity cycle because the released antigens from apoptotic cells exist in lymph nodes and stimulate T and B cells efficiently (**Figure 1** and **Figure 6**). Although the validation of the MUSCAT assay has not yet been completed by positive control of antibodies, this panel showed successful immune monitoring by detecting upregulation of several antibodies in the high responders by Ad-REIC cancer gene therapy. It is crucial for the diagnostic use of the MUSCAT assay that antigen spreading be observed before tumor regression. Baseline analysis of these autoantibodies showed individual variations in ten healthy donors (**Figure 6A**) although it is unclear whether these variations reflect the immune history of the individuals. However, monitoring autoantibody changes during therapy will evaluate the generation of cancer-immunity cycle and autoimmune responses. The concept of immunologically “hot” or “cold” from the outcome of immune checkpoint inhibitor therapy is widely accepted today, and the drug discovery to achieve “cold-to-hot” tumor conversion is a great challenge (57). Thus, a tool to monitor this conversion is critical. A highly accurate and quantitative autoantibody monitoring system will be an excellent tool for developing a therapeutic strategy to overcome refractory cancer.

## Data Availability Statement

The raw data supporting the conclusions of this article will be made available by the authors, without undue reservation.

## Ethics Statement

The studies involving human participants were reviewed and approved by Okayama University Hospital. The patients/participants provided their written informed consent to participate in this study.

## Author Contributions

AM, KK, and JF conceived the project. AM, TH, MM, and RK performed the experiments. AM and JF prepared the manuscript. HK analyzed the clinical data. All authors contributed to the article and approved the submitted version.

## Funding

This work is partially supported by JST START Grant Number JPMJST1918 (JF) and supported by Science and Technology Promotion grants (2019–2021) in Okayama Prefecture, Japan.

## Conflict of Interest

Okayama University and Medinet Co. Ltd. are holding patents on the method for MUSCAT assay.

The authors declare that the research was conducted in the absence of any commercial or financial relationships that could be construed as a potential conflict of interest.

## Publisher’s Note

All claims expressed in this article are solely those of the authors and do not necessarily represent those of their affiliated organizations, or those of the publisher, the editors and the reviewers. Any product that may be evaluated in this article, or claim that may be made by its manufacturer, is not guaranteed or endorsed by the publisher.

## Supplementary Material

Supplementary Figure 1 Preparation of recombinant antigens.

Supplementary Figure 2 XAGE-1 variants and immune reactivity of endogenous protein.

## References

[1] Lohmueller J Finn OJ. Current Modalities in Cancer Immunotherapy: Immunomodulatory Antibodies, Cars and Vaccines. Pharmacol Ther (2017) 178:31–47. doi: 10.1016/j.pharmthera.2017.03.008 28322974 PMC5600680

[2] Chen DS Mellman I. Oncology Meets Immunology: The Cancer-Immunity Cycle. Immunity (2013) 39(1):1–10. doi: 10.1016/j.immuni.2013.07.012 23890059

[3] Gajewski TF Schreiber H Fu YX. Innate and Adaptive Immune Cells in the Tumor Microenvironment. Nat Immunol (2013) 14(10):1014–22. doi: 10.1038/ni.2703 PMC4118725 24048123

[4] Jeanbart L Swartz MA. Engineering Opportunities in Cancer Immunotherapy. Proc Natl Acad Sci USA (2015) 112(47):14467–72. doi: 10.1073/pnas.1508516112 PMC4664348 26598681

[5] Nishikawa H Sakaguchi S. Regulatory T Cells in Cancer Immunotherapy. Curr Opin Immunol (2014) 27:1–7. doi: 10.1016/j.coi.2013.12.005 24413387

[6] Salemme V Centonze G Cavallo F Defilippi P Conti L. The Crosstalk Between Tumor Cells and the Immune Microenvironment in Breast Cancer: Implications for Immunotherapy. Front Oncol (2021) 11:610303. doi: 10.3389/fonc.2021.610303 33777750 PMC7991834

[7] Karasaki T Nagayama K Kuwano H Nitadori JI Sato M Anraku M. An Immunogram for the Cancer-Immunity Cycle: Towards Personalized Immunotherapy of Lung Cancer. J Thorac Oncol (2017) 12(5):791–803. doi: 10.1016/j.jtho.2017.01.005 28088513

[8] Kobayashi Y Kushihara Y Saito N Yamaguchi S Kakimi K. A Novel Scoring Method Based on Rna-Seq Immunograms Describing Individual Cancer-Immunity Interactions. Cancer Sci (2020) 111(11):4031–40. doi: 10.1111/cas.14621 PMC7648030 32810311

[9] Aptsiauri N Cabrera T Garcia-Lora A Lopez-Nevot MA Ruiz-Cabello F Garrido F. Mhc Class I Antigens and Immune Surveillance in Transformed Cells. Int Rev Cytol (2007) 256:139–89. doi: 10.1016/S0074-7696(07)56005-5 17241907

[10] Cheever MA Allison JP Ferris AS Finn OJ Hastings BM Hecht TT. The Prioritization of Cancer Antigens: A National Cancer Institute Pilot Project for the Acceleration of Translational Research. Clin Cancer Res (2009) 15(17):5323–37. doi: 10.1158/1078-0432.CCR-09-0737 PMC5779623 19723653

[11] Scanlan MJ Gure AO Jungbluth AA Old LJ Chen YT. Cancer/Testis Antigens: An Expanding Family of Targets for Cancer Immunotherapy. Immunol Rev (2002) 188:22–32. doi: 10.1034/j.1600-065x.2002.18803.x 12445278

[12] Scanlan MJ Simpson AJ Old LJ. The Cancer/Testis Genes: Review, Standardization, and Commentary. Cancer Immun (2004) 4:1. 14738373

[13] Tran E Turcotte S Gros A Robbins PF Lu YC Dudley ME. Cancer Immunotherapy Based on Mutation-Specific Cd4+ T Cells in a Patient With Epithelial Cancer. Science (2014) 344(6184):641–5. doi: 10.1126/science.1251102 PMC6686185 24812403

[14] Yadav M Jhunjhunwala S Phung QT Lupardus P Tanguay J Bumbaca S. Predicting Immunogenic Tumour Mutations by Combining Mass Spectrometry and Exome Sequencing. Nature (2014) 515(7528):572–6. doi: 10.1038/nature14001 25428506

[15] Alexandrov LB Nik-Zainal S Wedge DC Aparicio SA Behjati S Biankin AV. Signatures of Mutational Processes in Human Cancer. Nature (2013) 500(7463):415–21. doi: 10.1038/nature12477 PMC3776390 23945592

[16] Ohue Y Wada H Oka M Nakayama E. Antibody Response to Cancer/Testis (Ct) Antigens: A Prognostic Marker in Cancer Patients. Oncoimmunology (2014) 3(11):e970032. doi: 10.4161/21624011.2014.970032 25941600 PMC4292217

[17] Ohue Y Kurose K Mizote Y Matsumoto H Nishio Y Isobe M. Prolongation of Overall Survival in Advanced Lung Adenocarcinoma Patients With the Xage1 (Gaged2a) Antibody. Clin Cancer Res (2014) 20(19):5052–63. doi: 10.1158/1078-0432.CCR-14-0742 25124687

[18] Ohue Y Kurose K Karasaki T Isobe M Yamaoka T Futami J. Serum Antibody Against Ny-Eso-1 and Xage1 Antigens Potentially Predicts Clinical Responses to Anti-Pd-1 Therapy in Non-Small-Cell Lung Cancer. J Thorac Oncol (2019) 14(12):2071–83. doi: 10.1016/j.jtho.2019.08.008 31449889

[19] Gnjatic S Ritter E Büchler MW Giese NA Brors B Frei C. Seromic Profiling of Ovarian and Pancreatic Cancer. Proc Natl Acad Sci USA (2010) 107(11):5088–93. doi: 10.1073/pnas.0914213107 PMC2841879 20194765

[20] Gottschalk S Yu F Ji M Kakarla S Song XT. A Vaccine That Co-Targets Tumor Cells and Cancer Associated Fibroblasts Results in Enhanced Antitumor Activity by Inducing Antigen Spreading. PLoS One (2013) 8(12):e82658. doi: 10.1371/journal.pone.0082658 24349329 PMC3861387

[21] Nesslinger NJ Ng A Tsang KY Ferrara T Schlom J Gulley JL. A Viral Vaccine Encoding Prostate-Specific Antigen Induces Antigen Spreading to a Common Set of Self-Proteins in Prostate Cancer Patients. Clin Cancer Res (2010) 16(15):4046–56. doi: 10.1158/1078-0432.CCR-10-0948 PMC2912964 20562209

[22] Brossart P. The Role of Antigen Spreading in the Efficacy of Immunotherapies. Clin Cancer Res (2020) 26(17):4442–7. doi: 10.1158/1078-0432.CCR-20-0305 32357962

[23] Gupta A Nuber N Esslinger C Wittenbrink M Treder M Landshammer A. A Novel Human-Derived Antibody Against Ny-Eso-1 Improves the Efficacy of Chemotherapy. Cancer Immun (2013) 13:3. 23390374 PMC3559191

[24] de Moel EC Rozeman EA Kapiteijn EH Verdegaal EME Grummels A Bakker JA. Autoantibody Development Under Treatment With Immune-Checkpoint Inhibitors. Cancer Immunol Res (2019) 7(1):6–11. doi: 10.1158/2326-6066.CIR-18-0245 30425107

[25] Sakai Y Kurose K Sakaeda K Abo H Atarashi Y Ide N. A Novel Automated Immunoassay for Serum Ny-Eso-1 and Xage1 Antibodies in Combinatory Prediction of Response to Anti-Programmed Cell Death-1 Therapy in Non-Small-Cell Lung Cancer. Clin Chim Acta (2021) 519:51–9. doi: 10.1016/j.cca.2021.04.008 33865813

[26] Ohue Y Kurose K Karasaki T Isobe M Yamaoka T Futami J. Serum Antibody Against Ny-Eso-1 and Xage1 Antigens Potentially Predicts Clinical Responses to Anti-Programmed Cell Death-1 Therapy in Nsclc. J Thorac Oncol (2019) 14(12):2071–83. doi: 10.1016/j.jtho.2019.08.008 31449889

[27] DiLillo DJ Ravetch JV. Differential Fc-Receptor Engagement Drives an Anti-Tumor Vaccinal Effect. Cell (2015) 161(5):1035–45. doi: 10.1016/j.cell.2015.04.016 PMC4441863 25976835

[28] Kawabata R Wada H Isobe M Saika T Sato S Uenaka A. Antibody Response Against Ny-Eso-1 in Chp-Ny-Eso-1 Vaccinated Patients. Int J Cancer (2007) 120(10):2178–84. doi: 10.1002/ijc.22583 17278093

[29] Ohue Y Eikawa S Okazaki N Mizote Y Isobe M Uenaka A. Spontaneous Antibody, and Cd4 and Cd8 T-Cell Responses Against Xage-1b (Gaged2a) in Non-Small Cell Lung Cancer Patients. Int J Cancer (2012) 131(5):E649–58. doi: 10.1002/ijc.27359 22109656

[30] Ahmadi H Shogen K Fujita K Honjo T Kakimi K Futami J. Unusual Aggregation Property of Recombinantly Expressed Cancer-Testis Antigens in Mammalian Cells. J Biochem (2021) 170(3):435–43. doi: 10.1093/jb/mvab081 34247245

[31] Rajagopalan K Mooney SM Parekh N Getzenberg RH Kulkarni P. A Majority of the Cancer/Testis Antigens Are Intrinsically Disordered Proteins. J Cell Biochem (2011) 112(11):3256–67. doi: 10.1002/jcb.23252 PMC3214731 21748782

[32] Tompa P Csermely P. The Role of Structural Disorder in the Function of Rna and Protein Chaperones. FASEB J (2004) 18(11):1169–75. doi: 10.1096/fj.04-1584rev 15284216

[33] Futami J Nonomura H Kido M Niidoi N Fujieda N Hosoi A. Sensitive Multiplexed Quantitative Analysis of Autoantibodies to Cancer Antigens With Chemically S-Cationized Full-Length and Water-Soluble Denatured Proteins. Bioconjug Chem (2015) 26(10):2076–84. doi: 10.1021/acs.bioconjchem.5b00328 26355635

[34] Kimura S Imamura K Futami J. A Suitable and Effective Stepwise Oxidative Refolding Procedure for Highly-Cationic Tetrameric Avidin in Nucleic Acid Free Conditions. Biotechnol Prog (2020) 36(5):e3031. doi: 10.1002/btpr.3031 32463160

[35] Futami M Nakano T Yasunaga M Makihara M Asama T Hagihara Y. Enhanced in-Cell Folding of Reversibly Cationized Transcription Factor Using Amphipathic Peptide. J Biosci Bioeng (2017) 123(4):419–24. doi: 10.1016/j.jbiosc.2016.11.011 28110958

[36] Futami J Miyamoto A Hagimoto A Suzuki S Futami M Tada H. Evaluation of Irreversible Protein Thermal Inactivation Caused by Breakage of Disulphide Bonds Using Methanethiosulphonate. Sci Rep (2017) 7(1):12471. doi: 10.1038/s41598-017-12748-y 28963503 PMC5622167

[37] Bjerre M Hansen TK Flyvbjerg A Tønnesen E. Simultaneous Detection of Porcine Cytokines by Multiplex Analysis: Development of Magnetic Bioplex Assay. Vet Immunol Immunopathol (2009) 130(1-2):53–8. doi: 10.1016/j.vetimm.2009.01.007 19230983

[38] Ramaraj T Angel T Dratz EA Jesaitis AJ Mumey B. Antigen-Antibody Interface Properties: Composition, Residue Interactions, and Features of 53 Non-Redundant Structures. Biochim Biophys Acta (2012) 1824(3):520–32. doi: 10.1016/j.bbapap.2011.12.007 PMC3443979 22246133

[39] Kumon H Sasaki K Ariyoshi Y Sadahira T Ebara S Hiraki T. Ad-Reic Gene Therapy: Promising Results in a Patient With Metastatic Crpc Following Chemotherapy. Clin Med Insights Oncol (2015) 9:31–8. doi: 10.4137/CMO.S23252 PMC4373706 25861236

[40] Kumon H Ariyoshi Y Sasaki K Sadahira T Araki M Ebara S. Adenovirus Vector Carrying Reic/Dkk-3 Gene: Neoadjuvant Intraprostatic Injection for High-Risk Localized Prostate Cancer Undergoing Radical Prostatectomy. Cancer Gene Ther (2016) 23(11):400–9. doi: 10.1038/cgt.2016.53 PMC5116477 27767086

[41] Abarzua F Sakaguchi M Takaishi M Nasu Y Kurose K Ebara S. Adenovirus-Mediated Overexpression of Reic/Dkk-3 Selectively Induces Apoptosis in Human Prostate Cancer Cells Through Activation of C-Jun-Nh2-Kinase. Cancer Res (2005) 65(21):9617–22. doi: 10.1158/0008-5472.CAN-05-0829 16266978

[42] Sakaguchi M Kataoka K Abarzua F Tanimoto R Watanabe M Murata H. Overexpression of Reic/Dkk-3 in Normal Fibroblasts Suppresses Tumor Growth *Via* Induction of Interleukin-7. J Biol Chem (2009) 284(21):14236–44. doi: 10.1074/jbc.M808002200 PMC2682872 19279003

[43] Watanabe M Kashiwakura Y Huang P Ochiai K Futami J Li SA. Immunological Aspects of Reic/Dkk-3 in Monocyte Differentiation and Tumor Regression. Int J Oncol (2009) 34(3):657–63. doi: 10.3892/ijo_00000191 19212670

[44] Kinoshita R Watanabe M Huang P Li SA Sakaguchi M Kumon H. The Cysteine-Rich Core Domain of Reic/Dkk-3 Is Critical for Its Effect on Monocyte Differentiation and Tumor Regression. Oncol Rep (2015) 33(6):2908–14. doi: 10.3892/or.2015.3885 25823913

[45] Steinbuch M Audran R. The Isolation of Igg From Mammalian Sera With the Aid of Caprylic Acid. Arch Biochem Biophys (1969) 134(2):279–84. doi: 10.1016/0003-9861(69)90285-9 4982185

[46] Griffiths EA Srivastava P Matsuzaki J Brumberger Z Wang ES Kocent J. Ny-Eso-1 Vaccination in Combination With Decitabine Induces Antigen-Specific T-Lymphocyte Responses in Patients With Myelodysplastic Syndrome. Clin Cancer Res (2018) 24(5):1019–29. doi: 10.1158/1078-0432.CCR-17-1792 PMC5844797 28947565

[47] Sato S Noguchi Y Ohara N Uenaka A Shimono M Nakagawa K. Identification of Xage-1 Isoforms: Predominant Expression of Xage-1b in Testis and Tumors. Cancer Immun (2007) 7:5. 17335148 PMC2935747

[48] Nakagawa K Noguchi Y Uenaka A Sato S Okumura H Tanaka M. Xage-1 Expression in Non-Small Cell Lung Cancer and Antibody Response in Patients. Clin Cancer Res (2005) 11(15):5496–503. doi: 10.1158/1078-0432.CCR-05-0216 16061866

[49] Allison KH Sledge GW. Heterogeneity and Cancer. Oncology (Williston Park) (2014) 28(9):772–8. 25224475

[50] Wang C Yang J Luo H Wang K Wang Y Xiao ZX. Cancertracer: A Curated Database for Intrapatient Tumor Heterogeneity. Nucleic Acids Res (2020) 48(D1):D797–806. doi: 10.1093/nar/gkz1061 PMC7145559 31701131

[51] Correia IR. Stability of Igg Isotypes in Serum. MAbs (2010) 2(3):221–32. doi: 10.4161/mabs.2.3.11788 PMC2881250 20404539

[52] Ettinger R Sims GP Fairhurst AM Robbins R da Silva YS Spolski R. Il-21 Induces Differentiation of Human Naive and Memory B Cells Into Antibody-Secreting Plasma Cells. J Immunol (2005) 175(12):7867–79. doi: 10.4049/jimmunol.175.12.7867 16339522

[53] Chiappinelli KB Zahnow CA Ahuja N Baylin SB. Combining Epigenetic and Immunotherapy to Combat Cancer. Cancer Res (2016) 76(7):1683–9. doi: 10.1158/0008-5472.CAN-15-2125 PMC4873370 26988985

[54] Rosenthal R Cadieux EL Salgado R Bakir MA Moore DA Hiley CT. Neoantigen-Directed Immune Escape in Lung Cancer Evolution. Nature (2019) 567(7749):479–85. doi: 10.1038/s41586-019-1032-7 PMC6954100 30894752

[55] Yan X Zhao Y Liu Y Yang Q Dong L Wu Z. Case Report: Low-Dose Decitabine Plus Anti-Pd-1 Inhibitor Camrelizumab for Previously Treated Advanced Metastatic Non-Small Cell Lung Cancer. Front Oncol (2020) 10:558572. doi: 10.3389/fonc.2020.558572 33194624 PMC7649792

[56] Woloszynska-Read A Mhawech-Fauceglia P Yu J Odunsi K Karpf AR. Intertumor and Intratumor Ny-Eso-1 Expression Heterogeneity Is Associated With Promoter-Specific and Global DNA Methylation Status in Ovarian Cancer. Clin Cancer Res (2008) 14(11):3283–90. doi: 10.1158/1078-0432.CCR-07-5279 PMC2835568 18519754

[57] Liu YT Sun ZJ. Turning Cold Tumors Into Hot Tumors by Improving T-Cell Infiltration. Theranostics (2021) 11(11):5365–86. doi: 10.7150/thno.58390 PMC8039952 33859752

