# Supplementary material for: Engineering Cancer/Testis Antigens With Reversible S-Cationization to Evaluate Antigen Spreading
Source: Front Oncol. 2022 May 4;12:869393. doi: 10.3389/fonc.2022.869393 (PMC9115381; doi:10.3389/fonc.2022.869393)
Supplement: Supplementary Figure 1 — Preparation of recombinant antigens. [file DataSheet_1.docx]

Supplementary Material

## Supplementary Figure 1


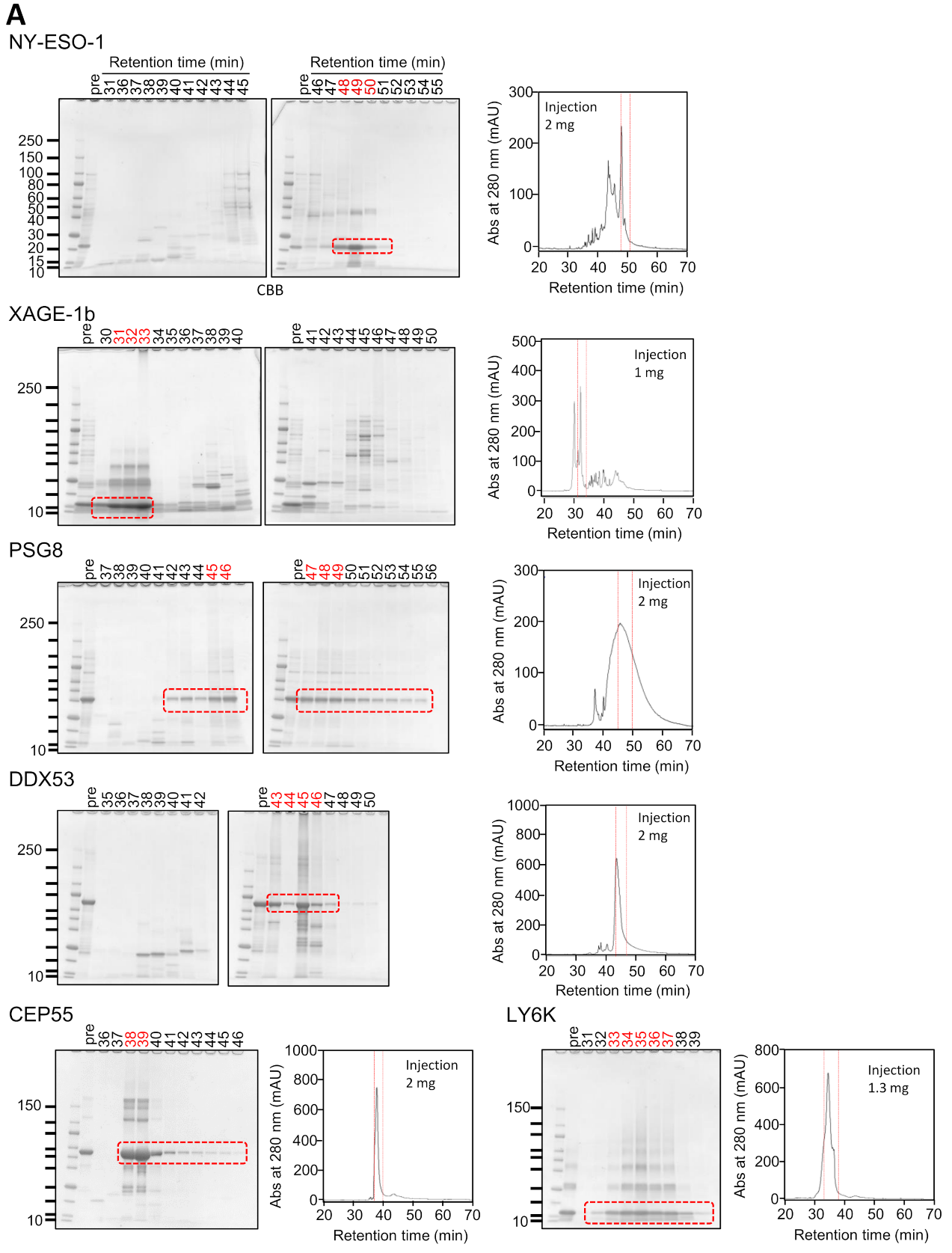


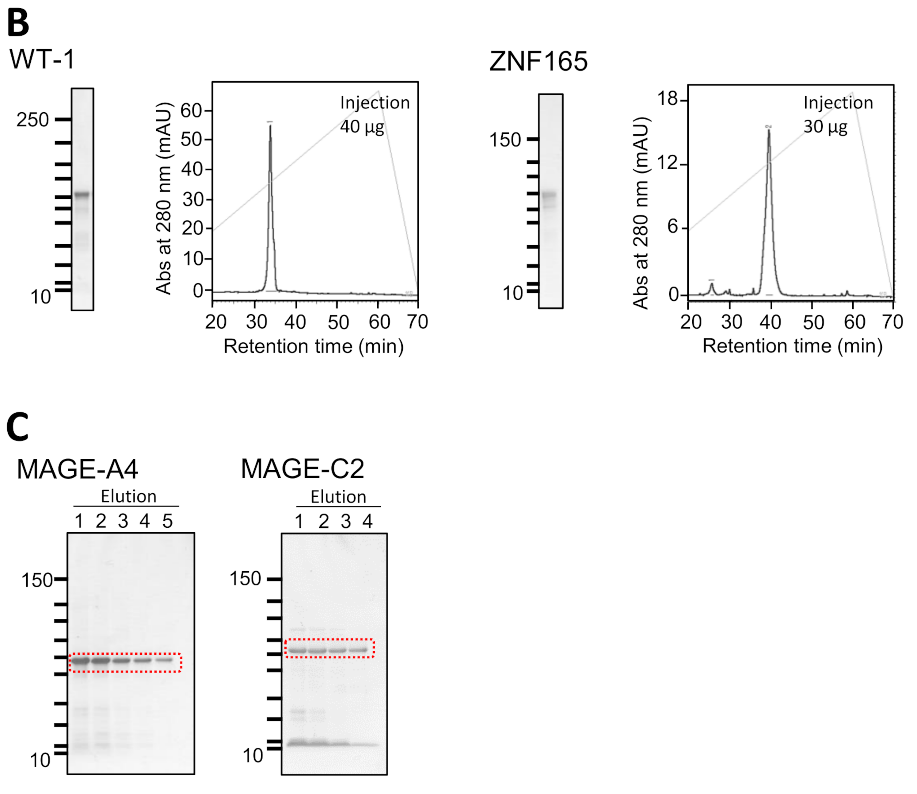


**Supplementary Figure 1.** Preparation of recombinant antigens.

(A) Chromatographic pattern and SDS-PAGE results for six TAPS antigens. The red dotted boxes indicate the area containing the antigens. The fractions highlighted in red were pooled before immobilization on Bio-Plex Pro™ Magnetic COOH Beads (B) Chromatographic pattern and SDS-PAGE results for TAPS-WT-1 and TAPS-ZNF165, both samples exhibited greater than 95% purity. (C) SDS-PAGE results for MAGE-A4 and MAGE-C2, both were purified by IMAC. The red dotted boxes indicate the area containing the antigens. Samples shown in (B) and (C) were immobilized to Bio-Plex Pro™ Magnetic COOH Beads without purification by a reversed-phase HPLC column.

## Supplementary Figure 2


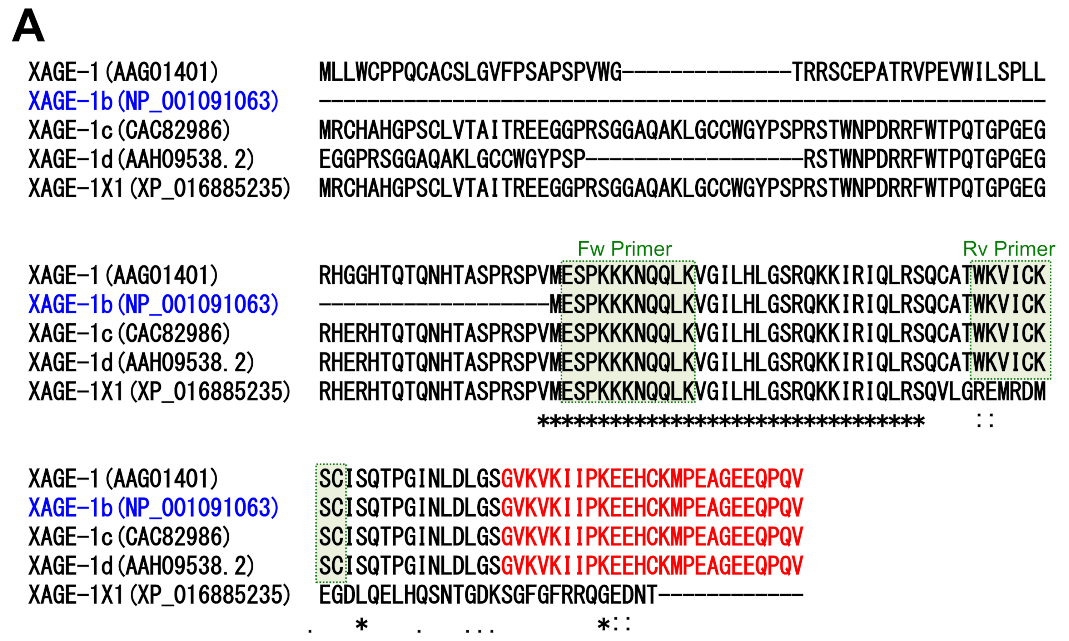


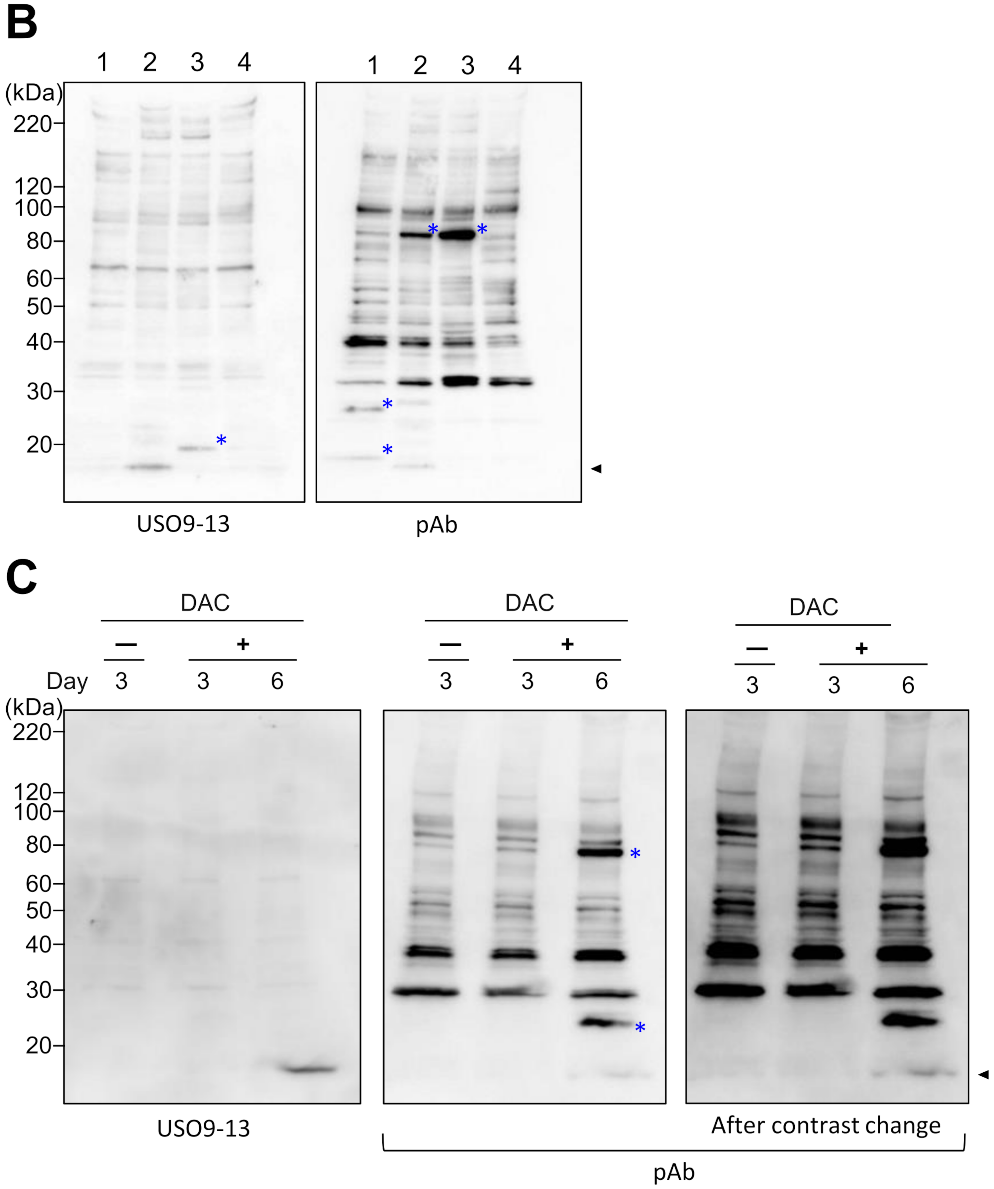


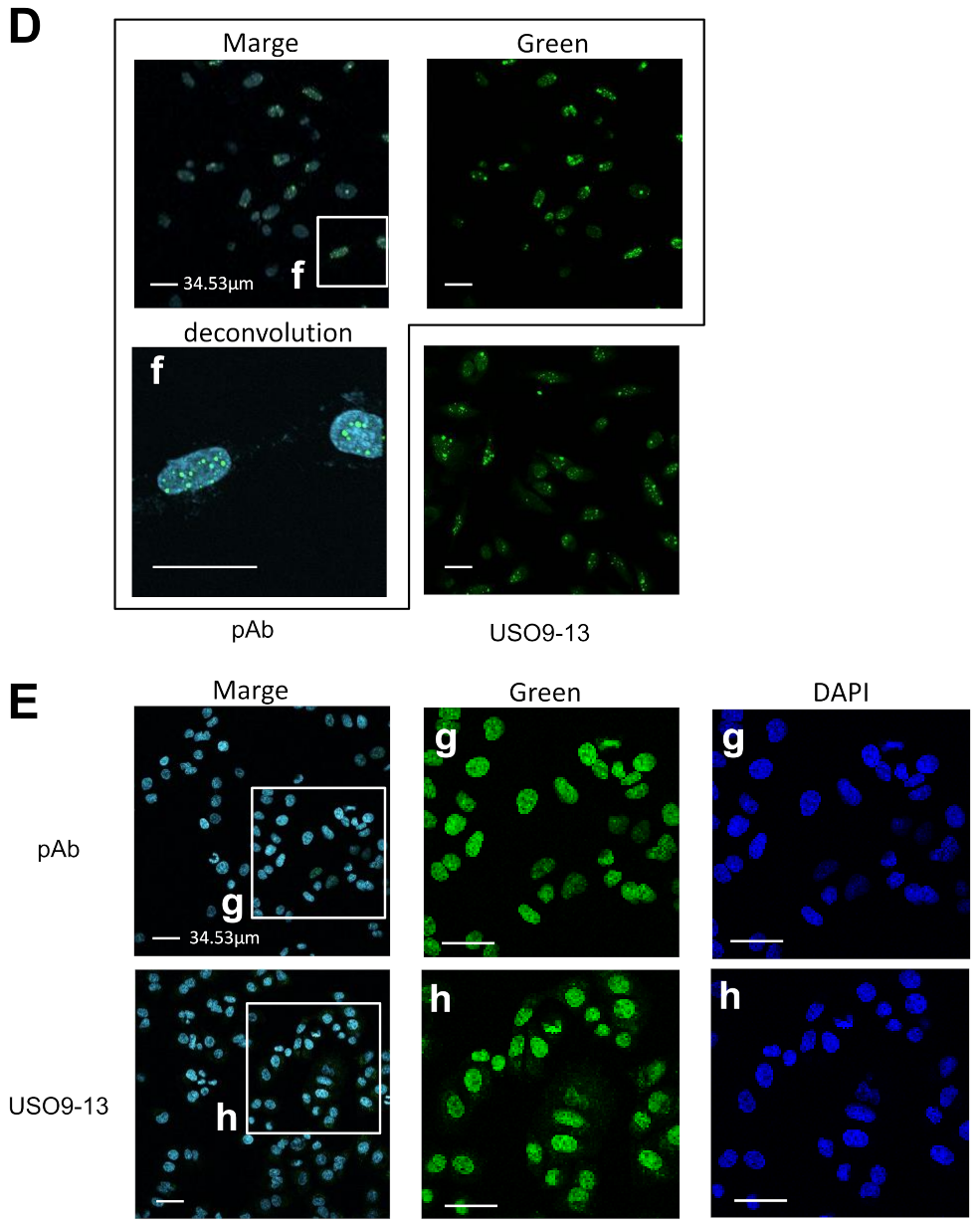


**Supplementary Figure 2.** XAGE-1 variants and immune reactivity of endogenous protein.

(A) Protein sequence alignment of XAGE-1, XAGE-1b, XAGE-1c, XAGE-1d, and XAGE-1X1 indicating the primer binding sites for RT-PCR (green box). (B) Detection of endogenous antigens in cell lysates, lane 1, NCI-H1299; lane 2, NCI-H1975; lane 3, A549; lane 4, HeLa S3 cells. Cell lysates were analyzed by western blotting, using XAGE-1b monoclonal antibody (USO9-13) or polyclonal antibody (pAb). * indicates the presumed XAGE-1 variants. (C) Detection of induced XAGE-1b expression protein by western blotting, using authorized XAGE-1b monoclonal antibody (USO9-13) or polyclonal antibody (pAb), in cell lysates of SK-OV-3 treated with 5 µM DAC. Samples were collected after three or six days of incubation with DAC. * indicates the presumed XAGE-1 variants. (D) Immunofluorescence staining of NCI-H1975 cells for nucleus (blue) and intracellular XAGE-1b (green) showing. XAGE-1b predominantly localized to the nucleus with characteristic dotted distribution. (E) Immunofluorescence staining of A549 cells for nucleus (blue) and intracellular XAGE-1b (green) indicating XAGE-1 variants localized to both the cytoplasm and nucleus.
